# Potential role of Fbxo22 in resistance to endocrine therapy in breast cancer with invasive lobular carcinoma

**DOI:** 10.1007/s10549-023-07209-2

**Published:** 2024-01-05

**Authors:** Saki Nakagawa, Minoru Miyashita, Ichiro Maeda, Atsushi Goda, Hiroshi Tada, Masakazu Amari, Yasuyuki Kojima, Koichiro Tsugawa, Yasuyo Ohi, Yasuaki Sagara, Miku Sato, Akiko Ebata, Narumi Harada-Shoji, Takashi Suzuki, Makoto Nakanishi, Tomohiko Ohta, Takanori Ishida

**Affiliations:** 1 Department of Breast and Endocrine Surgical Oncology, Tohoku University Graduate School of Medicine, 1-1 Seiryo-Machi, Aoba-Ku, Sendai, 980-8574 Japan; 2 Department of Breast Surgery, Osaki Citizen Hospital, Osaki, Japan; 3 Department of Pathology, Kitasato University Kitasato Institute Hospital, Tokyo, Japan; 4 Department of Pathology, Kitasato University School of Medicine, Sagamihara, Japan; 5 Department of Pathology, St. Marianna University School of Medicine, Kawasaki, Japan; 6 Department of Breast Surgery, Tohoku Kosai Hospital, Sendai, Japan; 7 Department of Breast and Endocrine Surgery, St. Marianna University School of Medicine, Kawasaki, Japan; 8 Department of Pathology, Hakuaikai Sagara Hospital, Kagoshima, Japan; 9 Department of Breast Surgical Oncology, Hakuaikai Sagara Hospital, Kagoshima, Japan; 10 Department of Pathology, Tohoku University Hospital, Sendai, Japan; 11 Division of Cancer Cell Biology, Institute of Medical Science, University of Tokyo, Tokyo, Japan; 12 Department of Translational Oncology, St. Marianna University Graduate School of Medicine, Kawasaki, Japan

**Keywords:** F-box protein 22 (Fbxo22), Breast cancer (BC), Invasive lobular carcinoma (ILC), Endocrine therapy, Resistance, Tamoxifen

## Abstract

**Background:**

Invasive lobular carcinoma (ILC) is distinct from invasive ductal carcinoma (IDC) in terms of their hormonal microenvironments that may require different therapeutic strategies. We previously reported that selective estrogen receptor modulator (SERM) function requires F-box protein 22 (Fbxo22). Here, we investigated the role of Fbxo22 as a potential biomarker contributing to the resistance to endocrine therapy in ILC.

**Methods:**

A total of 302 breast cancer (BC) patients including 150 ILC were recruited in the study. Fbxo22 expression and clinical information were analyzed to elucidate whether Fbxo22 negativity could be a prognostic factor or there were any correlations among clinical variables and SERM efficacy.

**Results:**

Fbxo22 negativity was significantly higher in ILC compared with IDC (58.0% vs. 27.0%, *P* < 0.001) and higher in postmenopausal patients than premenopausal patients (64.1% vs. 48.2%, *P* = 0.041). In the ILC cohort, Fbxo22-negative patients had poorer overall survival (OS) than Fbxo22-positive patients, with 10-year OS rates of 77.4% vs. 93.6% (*P* = 0.055). All patients treated with SERMs, Fbxo22 negativity resulted in a poorer outcome, with 10-year OS rates of 81.3% vs. 92.3% (*P* = 0.032). In multivariate analysis regarding recurrence-free survival (RFS) in ILC patients, Fbxo22 status was independently predictive of survival as well as lymph node metastasis.

**Conclusion:**

Fbxo22 negativity significantly impacts on survival in BC patients with IDC and ILC, and the disadvantage was enhanced among ILC postmenopausal women or patients treated with SERMs. The findings suggest that different therapeutic strategies might be needed according to the different histopathological types when considering adjuvant endocrine therapy.

## Introduction

Cancer is a major public health problem. Among them, breast cancer (BC) is the most frequently diagnosed cancer in women and remains an important cause of female cancer death worldwide [1]. Hormone receptor-positive BC, the most common subtype, expresses estrogen receptor (ER) and/or progesterone receptor (PgR), and endocrine therapy largely plays a pivotal role in decreasing disease recurrence and cancer death. Selective estrogen receptor modulators (SERMs) antagonizing ER activation by preventing cofactor binding [2] and aromatase inhibitors (AIs) blocking the conversion of androgen to estrogen [3] could contribute to an improvement in survival; however, therapeutic endocrine resistance has remained a major issue in the past decade in hormone receptor-positive BC [4].

A couple of mechanisms regarding endocrine resistance have been elucidated, and one of those reported is ESR1 mutation, which is found in approximately 30% of ER-positive BC patients previously treated with AIs [5]. As a suggestive molecule affecting the efficacy of endocrine therapy, F-box protein 22 (Fbxo22) is proposed to be an epigenetic multiplayer. Fbxo22 is a member of the F-box protein family that contains three functional domains: F-box and the F-box and intracellular signal transduction proteins FIST-N and FIST-C. It was originally reported to be a transcriptional target of p53 [6] and later to form a complex with KDM4A, whose degradation regulates histone H3 methylation at lysines 9 and 36 [7]. In a previous study, we identified a series of regulatory mechanisms controlling cofactor dynamics on ER and SERM function, whose activities require Fbxo22 [8]. The results notably illustrated that tamoxifen (TAM) released steroid receptor coactivator (SRC) and lysine demethylase 4B (KDM4B) from ER in a Fbxo22-dependent manner and that SRC released by TAM required Fbxo22 on almost all ER-SRC-bound enhancers and promoters. In vivo, TAM failed to prevent the growth of Fbxo22-depleted, ER-positive BCs. Among ER-positive and HER2-negative BCs with invasive ductal carcinoma (IDC), a low level of Fbxo22 in tumor tissues predicted a poorer outcome in our clinical cohort [8].

Invasive lobular carcinoma (ILC) is the second most frequently diagnosed histological subtype of invasive BC. The main morphological feature is characterized as a dysregulation of cell–cell adhesion, mainly derived from a lack of E-cadherin (CDH1) protein expression that is reported to be observed in 90% of ILC tumors [9, 10]. Morphological assessment and immunohistochemical analysis of CDH1 expression are often used to distinguish ILC from IDC. In clinical practice, the selection of therapeutic agents, including endocrine therapy and local treatment, such as radiotherapy, is not affected by the histopathological type [11, 12]. From the results of the BIG 1-98 trial, which was a phase III randomized controlled trial to test the efficacy of letrozole, an AI, compared to TAM in early BC, was associated with a significant reduction in overall survival (OS) compared to TAM among patients with ILC but not with IDC [13]. These findings were confirmed in another cohort of ILC patients in the ABCSG-8 trial comparing anastrozole, an AI, with TAM [14]. To date, no biological mechanism explaining the above reproducible results from clinical trials has been found in terms of the greater resistance to TAM in the ILC cohort than in the IDC cohort. Numerous studies have been performed to investigate the epidemiological and biological features as well as genomic profiles of ILCs. When exploring the profiles of ILC and IDC tumors, the results showed distinctive expression of genes associated with E-cadherin signaling, epithelial adhesion, and stromal rearrangement [15]. We also previously reported the original tumor microenvironment, including CAFs and the proliferation and maturation of intratumoral vessels, in ILC compared to IDC [16]. In addition to the tumor microenvironment, analyses of data from The Cancer Genome Atlas database show distinctive molecular aberrations in ILC compared with IDC, such as E-cadherin loss (66% vs. 3%), FOXA1 mutations (7% vs. 2%), and GATA3 mutations (5% vs. 20%) [17, 18]. However, the distinctive molecular profile affecting the responsiveness to endocrine therapy, specifically TAM, has not yet been clarified.

Although the academic community has extensively explored the biological difference between the two major morphological subtypes, namely, IDC and ILC, prior studies have failed to generate evidence to explain the distinctive efficacy of endocrine therapy between the two subtypes. Here, we identified Fbxo22 as a potential biomarker that contributes to the resistance of endocrine therapy, especially TAM, in ILC. Our study might contribute to the establishment of a new strategy for choosing different types of endocrine therapy according to BC subtypes.

## Materials and methods

### Clinicopathological features of patients and breast tissue specimens

This study included 140 nonmetastatic ER-positive and HER2-negative ILC patients who underwent primary surgical treatment at Tohoku University Hospital, Tohoku Kosai Hospital (Sendai, Japan), and Sagara Hospital (Kagoshima, Japan) between 2003 and 2013. Patient clinicopathological data were obtained from the three hospitals listed above, and the median follow-up period was 7.0 years. As a comparison, we used a set of paraffin-embedded core-needle biopsies from 163 ER-positive and HER2-negative patients in our previous study [8]. This cohort included 130 IDC patients, 10 ILC patients, and 22 patients with invasive carcinomas (mucinous carcinoma and other type). One ILC patient was excluded due to the lack of specimen for this study. These patients were consecutively treated for primary T2 (2–5 cm in diameter), and the relevant clinical data were obtained from patients underwent surgery at St. Marianna University Hospital (Kawasaki, Japan), between 2005 and 2009. The median follow-up period was 7.4 years in this comparative cohort.

These 302 patients were classified as having ER-positive and HER2-negative BC [16], and we determined high Ki-67 expression greater than 20% according to the St. Gallen International Expert Consensus recommendations of 2013 [19, 20]. Stage grouping was based on the TNM Classification of Malignant Tumors Eighth Edition by the Union for International Cancer Control (UICC) [21]. The tumor histological grade was determined according to the criteria of Elston and Ellis [22]. Histopathological diagnosis of ILC was confirmed by the absence of E-cadherin immunoreactivity. The protocol for this study was approved by the Ethics Committee at all institutions above.

### Immunohistochemistry and analysis of slides

**Fig. 1:**
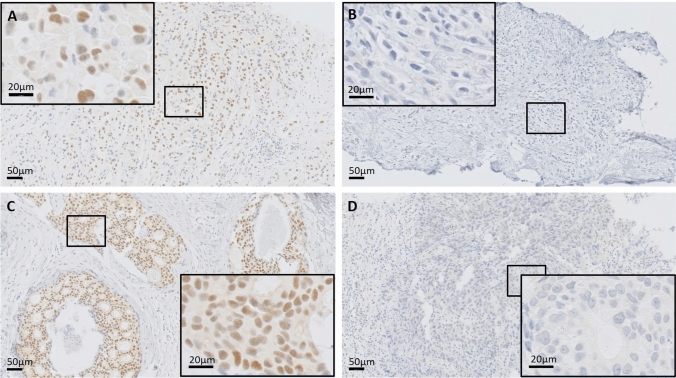
Representative immunohistochemical images of Fbxo22. Tumor cells showing moderate to strong nuclear staining are determined positive for Fbxo22. In ILC, discohesive tumor cells arrange in single file linear cords and invade the stroma. (**A**) Fbxo22-positive cells in ILC, (**B**) Fbxo22-negative cells in ILC. In IDC, tumor cells arrange in clusters with tubular structures. (**C**) Fbxo22-positive cells in IDC, (**D**) Fbxo22-negative cells in IDC

We performed immunohistochemistry for E-cadherin and Fbxo22 in this study to evaluate the expression of these antigens. The sections were deparaffinized in xylene and hydrated with graded alcohols and distilled water. Endogenous peroxidase activity was blocked by 3% hydrogen peroxidase for 10 min at room temperature (RT). For E-cadherin staining, we used a primary anti-E-cadherin antibody (4A2C7, Zymed) at a 1:400 dilution and detected it with a biotinylated rabbit anti-mouse antibody (Nichirei Bioscience) at a dilution of 1:100 for 30 min at RT and peroxidase-conjugated avidin (Nichirei Bioscience). For Fbxo22 staining, tissue sections were incubated with a primary anti-Fbxo22 antibody (GTX117774, GeneTex) at a 1:200 dilution and detected with an HRP-labeled polymer-conjugated secondary antibody (Histofine Simple Stain MAX PO, Nichirei). All the reacted sections were visualized using 3,3′-diaminobenzidine tetrahydrochloride. E-cadherin expression was evaluated in a blinded manner by two of the authors (Saki Nakagawa and Minoru Miyashita, Tohoku University Hospital), and Fbxo22 expression was evaluated by Ichiro Maeda and Yasushi Arizumi (St. Marianna University School of Medicine). In the evaluation of Fbxo22 expression, cancer tissues with one or more cells showing moderate or strong nuclear Fbxo22 staining of 100 cells examined were judged as Fbxo22 positive [8]. Furthermore, we divided Fbxo22-positive cases into three subgroups based on positivity of Fbxo22 (low, intermediate, high) and explored the association between the positivity and the clinical outcome. Patients whose proportion of Fbxo22-positive cells is 1–20% are categorized as low group, 21–50 as intermediate group, and 51% or more as high group (Fig. 1).

### Statistical analysis

The values for patient age are presented as the median and range. All data were evaluated using Student’s *t* test or the chi-square test based on whether the variable was continuous or categorical. A paired *t* test was used for the analysis of paired samples. The relationship between Fbxo22 status and various clinicopathological characteristics was evaluated with the chi-square test and Fisher’s exact test for categorical variables and Student’s *t* test for continuous variables. OS and recurrence-free survival (RFS) curves were constructed using the Kaplan‒Meier method, and the log-rank test was used to evaluate differences in the survival curves. A Cox proportional hazards regression model was used to estimate the hazard ratios (HRs) and 95% confidence intervals (CIs) of OS and RFS for each variable in the univariate and multivariate analyses. HRs and 95% CIs with two-sided *P* values are presented. IBM SPSS statistics version 27 (IBM, Armonk, NY, USA) was used for statistical analyses, and *P* values of less than 0.05 were considered statistically significant.

## Results

### Clinicopathological characteristics of ILC and IDC patients

This study included 302 ER-positive and HER2-negative invasive BC patients without distant metastases at the initial diagnosis. The clinicopathological characteristics of all patients included in this study are summarized in Table 1, and those of ILC patients are shown separately in Table 2. In the overall patient cohort, the median age was 55 years (range 29–91); the age distribution was not significantly different between the ILC and IDC cohorts. The median follow-up period was 7.3 years (1–201 months). In the ILC cohort, 58 patients were premenopausal females, but menstrual status was not provided for IDC patients. Of all 302 patients, 132 patients were positive for lymph node metastases, and 241 patients were classified as stage II or III (85 of 150 patients in the ILC cohort). Fifty-four patients in the ILC cohort received adjuvant chemotherapy, and 93 patients in the IDC cohort received neoadjuvant or adjuvant chemotherapy, including anthracycline followed by taxanes. In terms of adjuvant endocrine therapy, 106 of 302 patients received TAM only (60 in the ILC cohort), 153 patients received AIs only (73 in the ILC cohort), and 28 patients received both TAM and AI (6 in the ILC cohort) according to their menstrual status.

**Table 1:** Distribution of clinicopathological characteristics in all patients

	Total	Fbxo22 positive	Fbxo22 negative	*P* value
*N*	302	173 (57.3%)	129 (42.7%)	
*Age* (median 55, range 29–91)				
Age ≦ 54	147	94 (63.9%)	53 (36.1%)	
Age ≧ 55	155	79 (50.9%)	76 (49.1%)	***0.022***^a^
*Node*				
Positive	132	77 (58.3%)	55 (41.7%)	
Negative	170	96 (56.5%)	74 (43.5%)	0.745^a^
*Histological grade*				
I	142	88 (62.0%)	54 (38.0%)	
II–III	160	85 (53.1%)	75 (46.9%)	0.120^a^
*PgR*^b^				
Positive	230	141 (61.3%)	89 (38.7%)	
Negative	72	32 (44.4%)	40 (55.6%)	***0.012***^a^
*Ki-67-L*I^c^				
High	62	32 (51.6%)	30 (48.4%)	
Low	240	141 (58.8%)	99 (41.2%)	0.312^a^
*Stage*				
I	61	28 (45.9%)	33 (54.1%)	
II–III	241	145 (60.2%)	96 (39.8%)	0.059^d^
*Chemotherapy*				
No	155	87 (56.1%)	68 (43.9%)	
Yes	147	86 (58.5%)	61 (41.5%)	0.677^a^
*Hormone therapy*				
None	8 (2.6%)	4	4	
TAM	106 (35.1%)	65	41	
AI	153 (50.8%)	85	68	
Both (SERM and AI)	28 (9.2%)	16	12	
Others (TOR)	7 (2.3%)	3	4	0.872^b^
*Histology*^e^				
IDC	130	95 (73.1%)	35 (26.9%)	
ILC	150	63 (42.0%)	87 (58.0%)	
Others	22	15 (68.1%)	7 (31.9%)	**<*0.001***^a^

Bold italics indicate that P values are less than 0.05

*P* values of less than 0.05 were considered statistically significant

*IDC* invasive ductal carcinoma, *ILC* invasive lobular carcinoma, *PgR* progesterone receptor, *TAM* tamoxifen, *AI* aromatase inhibitor, *SERM* selective estrogen receptor modulators, *TOR* toremifen

^a^Chi-square test

^b^PgR status 0% or <1%

^c^Ki-67-LI cut-off: 20%

^d^Fisher’s exact test

^e^Determined according to WHO 2012 classification

**Table 2:** Distribution of clinicopathological characteristics in ILC patients

	Total	Fbxo22 positive	Fbxo22 negative	*P* value
*N*	150	63 (42.0%)	87 (58.0%)	
*Age (median)*	57			
Premenopause	58	30 (51.8%)	28 (48.2%)	
Postmenopause	92	33 (35.9%)	59 (64.1%)	***0.041***^a^
*Node*				
Positive	47	17 (36.1%)	30 (63.9%)	
Negative	103	46 (44.7%)	57 (55.3%)	0.328^b^
*Histological grade*				
I	55	21 (38.1%)	34 (61.9%)	
II–III	95	42 (44.2%)	53 (55.8%)	0.471^b^
*PgR*^c^				
Positive	106	48 (45.3%)	58 (54.7%)	
Negative	44	15 (34.1%)	39 (65.9%)	0.206^b^
*Ki-67-LI*^d^				
High	11	4 (36.4%)	7 (63.6%)	
Low	139	59 (42.4%)	80 (57.6%)	0.476^a^
*Stage*				
I	65	30 (46.2%)	35 (53.8%)	
II–III	85	33 (38.8%)	52 (61.2%)	0.367^b^
*Chemotherapy*				
No	96	42 (43.8%)	54 (56.2%)	
Yes	54	21 (38.9%)	33 (61.1%)	0.563^b^
*Hormone therapy*				
None	5 (3.3%)	1	4	
TAM	60 (40.0%)	31	29	
AI	73 (48.7%)	27	46	
Both (SERM and AI)	6 (4.0%)	2	4	
Others (TOR)	6 (4.0%)	2	4	0.300^a^

Bold italics indicate that P values are less than 0.05

*P* values of less than 0.05 were considered statistically significant

*IDC* invasive ductal carcinoma, *ILC* invasive lobular carcinoma, *PgR* progesterone receptor, *TAM* tamoxifen, *AI* aromatase inhibitor, *SERM* selective estrogen receptor modulators, *TOR* toremifen

^a^Fisher’s exact test

^b^Chi-square test

^c^PgR status 0% or <1%

^d^Ki-67-LI cut-off: 20%

### Fbox22 negativity is significantly higher in ILC patients than in IDC patients

**Fig. 2:**
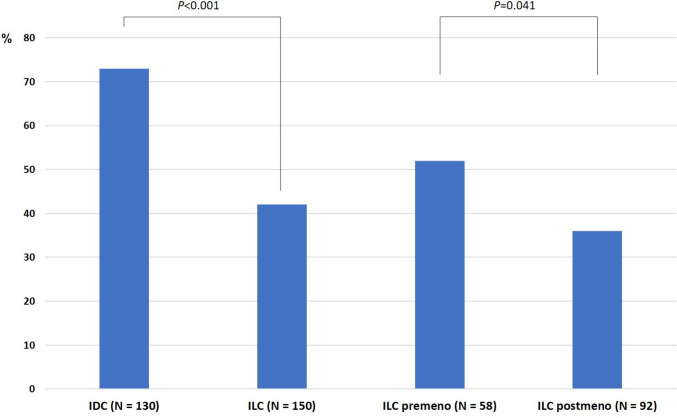
The proportion of Fbxo22-positive tumor (IDC vs. ILC, or premenopausal vs. postmenopausal among ILC). Sixty-three (42.0%) patients were determined to be positive for Fbxo22 expression in the ILC cohort (*N* = 150), which is significantly lower than that of the IDC cohort (the positivity rate: 73.0%, 95/130). In the ILC cohort, Fbxo22 positivity was lower in postmenopausal ILC patients (35.9%, 33/92) than in premenopausal ILC patients (51.8%, 30/58)

Of the 302 patients, 129 (42.7%) patients were determined to be negative for Fbxo22 expression (Table 1). Fbxo22 negativity was correlated with negative PgR status but not with high Ki-67 positivity, lymph node involvement, or tumor grade (Table 1). In the ILC cohort, 87 (58.0%) patients were determined to be negative for Fbxo22 expression (Fig. 2), which showed a significantly higher rate of negativity than that in the IDC cohort (26.9%, *P* < 0.001). In particular, Fbxo22 negativity was significantly higher in postmenopausal ILC patients (64.1%) than in premenopausal ILC patients (48.2%, *P* = 0.041) (Table 2 and Fig. 2). On the contrary, there were no correlations between Fbxo22 status and any other variables including PgR (Table 2).

### The lack of Fbxo22 expression is a poorer prognostic factor among the patients treated with SERMs

**Fig. 3:**
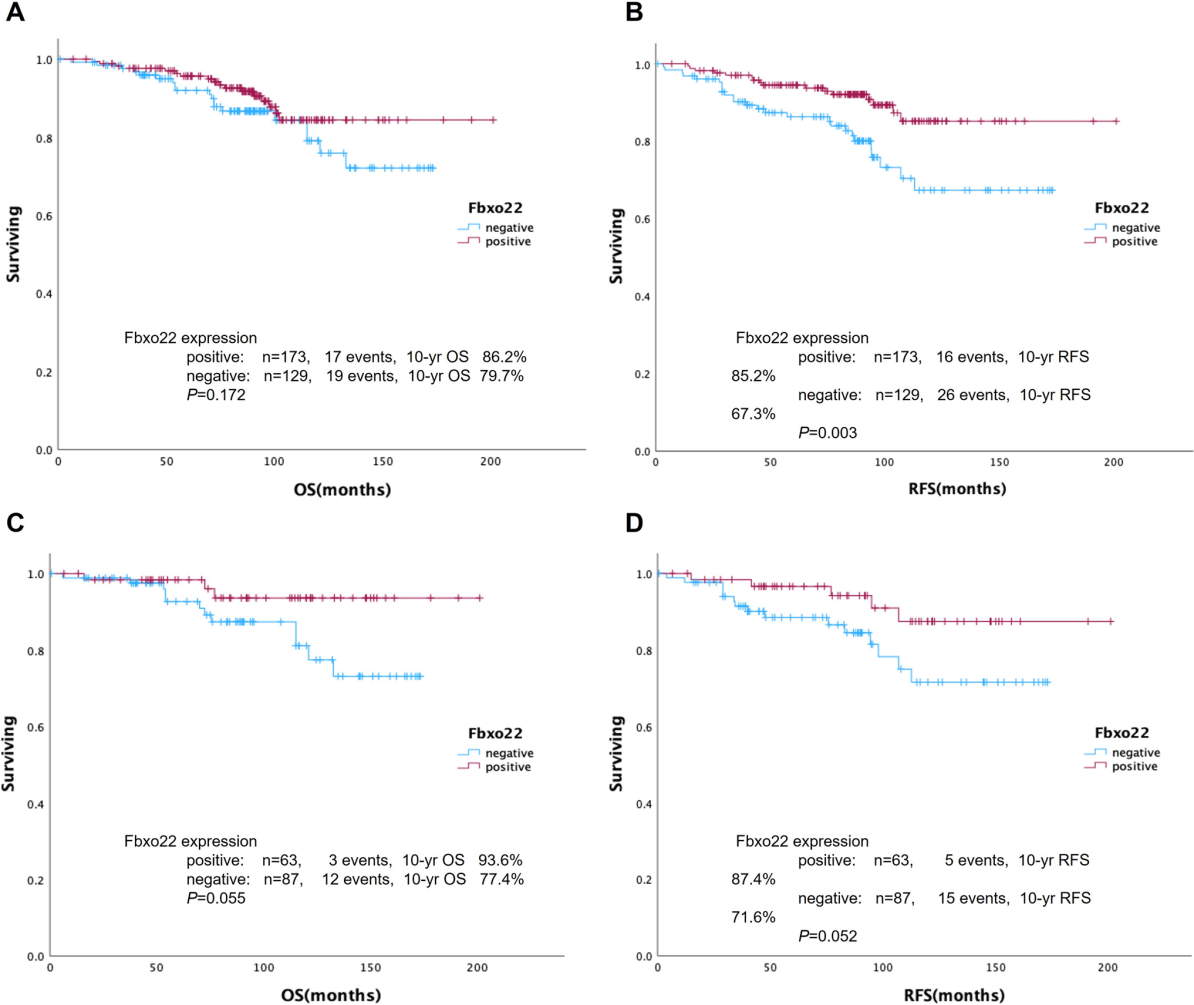
Kaplan‒Meier curves stratified by Fbxo22 protein expression in ER-positive/HER2-negative cases. (**A**) OS and (**B**) RFS in the entire cohort, (**C**) OS and (**D**) RFS in the ILC cohort. *P* values were calculated using the log-rank test. *P* values of less than 0.05 were considered statistically significant

To evaluate the associations between Fbxo22 expression and prognosis, we analyzed OS and RFS in all patients and in the ILC cohort (Fig. 3 and Table 3). In the overall patient cohort, 42 patients (20 of 150 ILC patients) had distant or locoregional recurrences within the median time of 44 months (3–113 months). In multivariate RFS analyses, Fbxo22 negativity and node positivity were independently predictive of poorer RFS. The clinical data indicated that the lack of Fbxo22 expression resulted in a poorer outcome regardless of ILC or IDC, low Ki-67 expression, node-negative status, low tumor grade, or treatment with TAM.

**Table 3:** Association between Fbxo22 expression or clinical variables and recurrence-free survival in all patients

Variables	Univariate			Multivariate		
	HR	95% CI	*P* value	HR	95% CI	*P* value
*Fbxo22*						
Positive	1.00	0.217, 0.754	***0.027***	1.00	0.206, 0.801	***0.009***
Negative	2.47			2.46		
*Node*						
Negative	1.00	1.269, 4.494	***0.007***	1.00	1.018, 4.364	***0.045***
Positive	2.39			2.11		
*PgR status*						
Positive	1.00	1.012, 1.904	***0.042***	1.00	0.955, 1.841	0.092
Negative	1.39			1.74		
*Ki-67-LI*						
Low	1.00	0.842, 1.725	0.307	1.00	0.700, 1.584	0.804
High	1.21			1.05		
*Stage*						
I	1.00	0.904, 7.133	0.077	1.00	0.529, 5.889	0.356
II, III	2.54			1.76		
*Histological grade*						
I	1.00	0.542, 1.833	0.992	1.00	0.482, 1.754	0.800
II, III	1.00			1.13		
*IDC or ILC*						
IDC	1.00	0.796, 1.472	0.613	1.00	0.723, 1.581	0.738
ILC	1.08			1.07		
*Treated with TAM*						
Yes	1.00	0.823, 1.515	0.477	1.00	0.674, 2.529	0.437
No	1.12			1.30		

Bold italics indicate that P values are less than 0.05

Estimated from Cox proportional hazards model. *P* values of less than 0.05 were considered statistically significant. Ki-67 Labeling Index cut-off was determined 20%

*PgR* progesterone receptor, *IDC* invasive ductal carcinoma, *ILC* invasive lobular carcinoma, *TAM* tamoxifen

The 10-year OS and 10-year RFS rates stratified by Fbxo22 expression in the overall patient cohort and in the ILC cohort are shown in Fig. 3A–D. In the overall patient cohort, Fbxo22-negative patients had poorer RFS than Fbxo22-positive patients, with 10-year RFS rates of 67.3% vs. 85.2% (*P* = 0.003), but there was no significant difference in the 10-year OS rate (79.7% vs. 86.2%, *P* = 0.172). In the separate ILC cohort, this tendency was preserved, with 10-year RFS rates of 71.6% vs. 87.4% (*P* = 0.052) and 10-year OS rates of 77.4% vs. 93.6% (*P* = 0.055). Furthermore, we explore the survival impact of Fbxo22 among PgR positive ILC cohort (*N* = 102) that is generally considered to have better outcome than PgR negative ILC cohort. Consequently, Fbxo22-negative patients tend to have poorer RFS and OS than Fbxo22-positive patients but not statistically significant (RFS; *P* = 0.190, OS; *P* = 0.187). In addition, comparing the clinical outcome among Fbxo22-positive patients (*N* = 173) based on the positivity of Fbxo22 (low, intermediate, or high), there was no significant difference of RFS and OS among low (*N* = 84), intermediate (*N* = 59), and high group (*N* = 30) (RFS; *P* = 0.691, OS; *P* = 0.146).

**Fig. 4:**
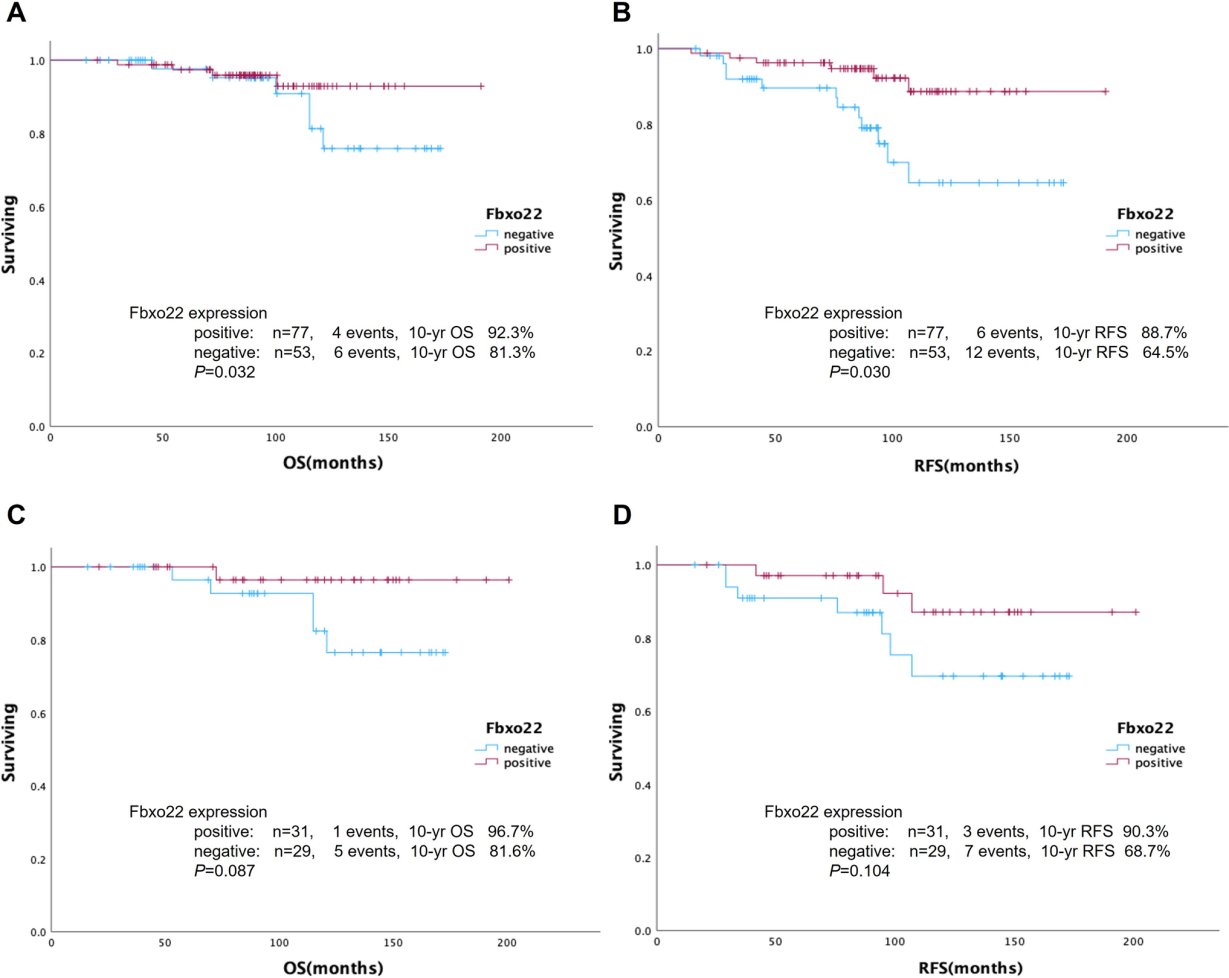
Kaplan‒Meier curves stratified by Fbxo22 protein expression in ER-positive/HER2-negative cases. (**A**) OS and (**B**) RFS in SERM-treated cases, (**C**) OS and (**D**) RFS in TAM-treated ILC cases. *P* values were calculated using the log-rank test. *P* values of less than 0.05 were considered statistically significant

In our previous study, low Fbxo22 expression was associated with SERM resistance, resulting in poorer outcomes in patients with ER-positive/HER2-negative IDC [8]. Thus, we analyzed the prognosis of all patients and patients in the ILC cohort treated with SERMs to assess whether Fbxo22 negativity could be a prognostic factor in these cohorts as well. The 10-year OS and 10-year RFS rates in these cohorts stratified by Fbxo22 expression are shown in Fig. 4A–D. In all patients treated with SERMs, the lack of Fbxo22 expression resulted in a poorer outcome, with 10-year OS rates of 81.3% vs. 92.3% (*P* = 0.032) and 10-year RFS rates of 64.5% vs. 88.7% (*P* = 0.030). This tendency was preserved in ILC patients, but there was no significant difference (10-year OS rates of 81.6% vs. 96.7% (*P* = 0.087) and 10-year RFS rates of 68.7% vs. 90.3% (*P* = 0.104)).

## Discussion

We first demonstrated that ER-positive BC patients negative for Fbxo22 expression had significantly worse survival than those positive for Fbxo22 expression in both IDC and ILC, which are the two major morphological types of BC. Furthermore, the survival disadvantage among BC patients with Fbxo22-negative tumors was maintained when BC patients treated with adjuvant TAM therapy. The real-world evidence from the present study strongly supports our finding that SRC released by TAM requires Fbxo22 on almost all ER SRC-bound enhancers and promoters, resulting in TAM failing to prevent the growth of Fbxo22-negative, ER-positive tumors in our previous study. These findings uncovering one of the mechanisms of endocrine resistance could highlight a potential strategy for overcoming cancer recurrence and death in ER-positive BC.

Although preclinical and translational research has been conducted focusing on ER-positive BC, specific systemic therapies targeting ILC, which account for 5–15% of all BCs, do not exist. The lack of evidence regarding treatment for ILC forces physicians to make a decision when treating ILC patients based on the evidence of clinical trials mainly including IDC patients. To better understand of the molecular features of ILC, which generally present as luminal A-like tumors, the TCGA research group performed a comprehensive molecular analysis of luminal A ILC compared to luminal A IDC [17]. Regarding ER activity, their analysis showed that FOXA1 and GATA3, major regulators of the ER transcriptional program [17], were differentially mutated in ILC compared to IDC, suggesting that different mechanisms of tumor progression relying on ER signaling exist between the two histological subtypes (FOXA1: 7% in ILC vs. 2% in IDC; GATA3: 5% in ILC vs. 13% in IDC). Arthur et al. previously reported that alterations in gene expression in response to letrozole were highly similar between responding ILC and IDC, namely, genes involved in proliferation were downregulated, whereas those involved in immune function and extracellular matrix remodeling were upregulated [15]. However, to our knowledge, there have been few previous studies on the molecular signature related to resistance to endocrine therapy in ILC patients.

Among the ILC cohort, 58.0% of patients had Fbxo22-negative tumors, which was significantly higher than that of patients in the IDC cohort (26.9%). Furthermore, the lower expression rate of Fbxo22 in ILC tumors is more highlighted when focusing on postmenopausal women; it is significantly different compared with that in premenopausal women. In 150 ILC patients, there were no clinicopathological factors associated with the expression level of Fbxo22 except for menopausal status (Table 2). One potential explanation regarding the difference in Fbxo22 negativity between ILC and IDC, especially ILC in postmenopausal women, might be the differential tumor microenvironment in terms of the estrogen signal transduction pathway between the two histological types. A previous study reported that the intratumoral concentrations of both estrone (E1) and estradiol (E2) were higher in luminal A IDC than in luminal A ILC [23]. In circumstances with low E1 and E2 levels, Fbxo22, as a negative regulator disassembling KDB4B, might not be required to maintain the homeostasis of ER-positive cancer cells, resulting in Fbxo22 being naturally downregulated in ILCs in postmenopausal women. The interaction between Fbxo22 and PgR should be discussed to clarify the biological feature specifically in ILC. Although Fbxo22 negativity is significantly associated with the status of PgR in the entire cohort (Table 1), the association was not found in ILC cohort (Table 2). From the result that Fbxo22-negative patients tend to have poorer outcome than Fbxo22-positive patients In PgR positive ILC cohort, Fbxo22 might affect the outcome of ILC patients regardless of PgR function. To the best of our knowledge, there was no previous research that investigated the interaction between Fbxo22 and PgR. Fbxo22 might indirectly affect the function of PgR through ER and the further research is required to elucidate how Fbxo22 affects the function of PgR.

Two large clinical trials indicated a greater benefit of adjuvant letrozole or anastrozole than TAM for patients with ILC but not for those with IDC [13, 14]. In the BIG 1-98 trial, the 8-year RFS estimate was 66% for TAM compared with 82% for letrozole in the ILC population with an HR of 0.48, whereas the HR was 0.80 in the IDC population (interaction *P* = 0.03). The 8-year OS estimates were 74% for TAM compared with 89% for letrozole in the ILC subset (HR: 0.40) and 84% for TAM and 88% for letrozole in the IDC subset, whereas the HR was 0.73 in the IDC population (interaction *P* = 0.45). In clinical practice, no guidelines have recommended the choice of an AI against TAM based on the histopathological type rather than tumor stage and toxic profile. All of the previous basic or clinical studies suggesting therapeutic resistance to TAM in ILC have a retrospective nature; therefore, the results must be carefully interpreted. However, our preclinical findings regarding Fbxo22 in postmenopausal women with ILC tumors consistently support the biological mechanism of resistance to TAM.

The incidence of ILC among all histopathological types is 5–15% [24, 25], which is the main reason why the biological clarification of ILC has not progressed compared with IDC. Our cohort including 150 patients with ILC is relatively large; however, one of the limitations of the study is the small sample size of ILC patients treated with TAM (*N* = 60), which might be inadequate to investigate the survival difference according to the status of Fbxo22 expression. Our findings should be verified with a larger cohort that includes postmenopausal women treated with TAM.

In conclusion, Fbxo22 negativity has a significant impact on survival in BC patients with IDC and ILC, and the disadvantage was enhanced among postmenopausal women with ILC, or patients treated with adjuvant TAM therapy. The findings suggest that different therapeutic strategies might be needed according to the different histopathological types when considering adjuvant endocrine therapy.

## Data Availability

The datasets generated during and/or analysed during the current study are not publicly available due to individual privacy but are available from the corresponding author on reasonable request.

## Declarations

### Registry and the Registration No. of the study/trial

N/A.

### Animal studies

N/A.
